# Study Design Indexing in Transition: A Focused Comparison of manual NLM Indexing vs. Transformer-based Automated Models

**DOI:** 10.64898/2026.06.03.26354854

**Published:** 2026-06-04

**Authors:** Puranjani Das, Jodi Schneider, Evan Mayo-Wilson, Halil Kilicoglu, Joe D. Menke, Dongin Nam, Kiran Ninan, Jean-Pierre Oberste, Ang Michael Troy, Xiangji Ying, Arthur W. Holt, Neil R. Smalheiser

## Abstract

**Objectives:**

Study design indexing of biomedical publications is crucial for evidence retrieval and synthesis. We sought to evaluate the accuracy and suitability of a transformer-based model (TM) for indexing clinical study designs, in comparison to National Library of Medicine (NLM) indexing. However, this is challenging for at least three reasons: First, to date, all automated systems have been trained and evaluated on manual NLM indexing assignments, itself subject to errors. Second, TM’s probabilistic predictive scores take into account uncertainty, and can be converted to TRUE/FALSE assignments in different ways depending on the needs of users, while NLM labels are categorical. Third, our goal (to tag articles only that exhibit a given design) differs from NLM which tags articles that both discuss as well as exhibit that design.

**Materials and Methods:**

Therefore, we carried out a limited evaluation of the TM model that focuses only on the articles that received the most confident predictions, that is, the highest scores that are almost certainly TRUE and the lowest scores that are almost certainly FALSE, but which disagreed with NLM assignments. This was performed both for articles published in 2016 (when NLM decisions were manual) and in 2025 (when NLM decisions were automated). To establish ground truth, dual annotators indexed the articles independently, following written definitions, for four prominent study designs—cohort, case-control, cross-sectional, and case report.

**Results:**

For three designs (case-control, case report, cross-sectional), the articles having the top 100 predictive TM scores (when NLM failed to assign that design) were judged to exhibit that design in the great majority (86-100%) of cases. Conversely, the articles having the lowest 100 predictive TM scores (when NLM did assign the study design) exhibited the design only in relatively few (0-21%) of cases. The most confident predictions of the TM model were highly accurate and not redundant with automated NLM indexing; the exception was cohort studies articles, in which both TM and NLM labels showed high error rates of both omission and commission.

**Discussion and Conclusion:**

TM may have value for identifying articles exhibiting study designs, which is especially important for clinical decision-making as well as systematic reviews and other evidence syntheses. NLM indexing of cohort studies cannot be regarded as a reliable gold standard for training or evaluation of automated systems, warranting efforts to create a new manually annotated corpus.

## Full Text Availability

The license terms selected by the author(s) for this preprint version do not permit archiving in PMC. The full text is available from the preprint server.

